# Chelidonic acid ameliorates *Aspergillus fumigatus* keratitis via antifungal and anti-inflammatory activities

**DOI:** 10.3389/fimmu.2026.1928802

**Published:** 2026-08-27

**Authors:** Wenting Liu, Qiang Xu, Zhen Liu, Jintao Sun, Xiaoyan Sun, Xiaomei Wan, Shasha Xue, Chengye Che, Shaoqi Tian

**Affiliations:** 1 Department of Ophthalmology, the Affiliated Hospital of Qingdao University, Qingdao, China; 2 Department of Orthopedic Surgery, the Affiliated Hospital of Qingdao University, Qingdao, China

**Keywords:** antifungal, anti-inflammatory, *Aspergillus fumigatus*, chelidonic acid, fungal keratitis

## Abstract

**Purpose:**

This study aimed to evaluate the therapeutic efficacy of chelidonic acid (CA) in *Aspergillus fumigatus* (*AF*)-induced keratitis and to elucidate the underlying molecular mechanisms.

**Methods:**

The safety of CA was assessed using a Cell Counting Kit-8 (CCK-8) assay, live-dead cell staining, cell scratch assay, corneal fluorescein sodium staining, and hematoxylin-eosin (HE) staining. The antifungal activity of CA was determined using minimum inhibitory concentration (MIC) assays, calcofluor white staining, propidium iodide (PI) staining, quantification of corneal fungal burden, periodic acid-Schiff (PAS) staining and quantitative real-time polymerase chain reaction (qRT-PCR). The therapeutic efficacy of CA was evaluated by slit-lamp biomicroscopy, corneal clinical scoring, HE staining, immunofluorescence staining, myeloperoxidase (MPO) measurement, and Transwell migration assay. The anti-inflammatory activity of CA was determined by qRT-PCR, western blotting, and immunofluorescence staining. The therapeutic efficacy of combined CA and natamycin treatment was assessed by slit-lamp examination, corneal clinical scoring, PAS staining, and HE staining.

**Results:**

5 mM CA exhibited no detectable cytotoxicity in RAW264.7 cells, human corneal epithelial cells (HCECs) or mouse corneas. CA exerted antifungal effects in *AF* keratitis. CA significantly ameliorated *AF* keratitis by suppressing neutrophil and macrophage infiltration. CA markedly downregulated the expression of pro-inflammatory cytokines, including interleukin-1β (IL-1β), IL-6, and tumor necrosis factor-α (TNF-α), and upregulated the expression of anti-inflammatory cytokine IL-10, through inhibition of the myeloid differentiation factor 88 (MyD88)/nuclear factor-kappa B (NF-κB) signaling pathway. Combined treatment with CA and natamycin further reduced clinical scores, fungal load, and inflammatory cell infiltration compared with monotherapy.

**Conclusions:**

CA exhibits both antifungal and anti-inflammatory activities in *AF* keratitis and represents a promising candidate for the treatment of fungal keratitis.

## 1 Introduction

Fungal keratitis (FK) is a severe ocular infection caused by pathogenic fungi and frequently results in profound visual impairment or irreversible vision loss (1). Common causative organisms include *Fusarium* and *Aspergillus* species (2). FK is particularly prevalent in developing countries, especially in tropical and subtropical agricultural regions (3). In recent years, its incidence has increased markedly, largely due to improper contact lens use, inappropriate corticosteroid administration, and a rising frequency of corneal trauma (4, 5). However, conventional antifungal therapies are limited by poor aqueous solubility, inadequate corneal penetration, ocular surface toxicity, and emerging drug resistance, often leading to suboptimal clinical outcomes (6). Therefore, the development of novel and effective therapeutic strategies for FK remains an urgent clinical need.

Following fungal invasion of the cornea, host pattern recognition receptors (PRRs) detect fungal pathogen-associated molecular patterns (PAMPs), thereby triggering innate immune responses (7). Activation of downstream intracellular signaling cascades induces robust production of pro-inflammatory cytokines, which recruit neutrophils and macrophages to the site of infection to eliminate fungal pathogens (8, 9). However, excessive or sustained inflammatory responses result in massive infiltration of inflammatory cells and overproduction of cytokines, leading to irreversible corneal injury (10). Therefore, ideal therapeutic agents for FK should combine antifungal efficacy with the ability to modulate excessive inflammation.

Chelidonic acid (CA; 4-oxo-4H-pyran-2,6-dicarboxylic acid) is a naturally occurring secondary metabolite predominantly found in Chelidonium majus (11–14). It exhibits diverse pharmacological activities, including analgesic, antibacterial, anti-inflammatory, antioxidant, anticancer, and osteogenic effects, and has been applied in various medicinal contexts (12, 15–17). Previous studies have shown that Chelidonium majus L. (Papaveraceae) possesses antiviral and antibacterial properties (18). CA levels increase in plants following fungal infection and significantly inhibit the mycelial growth of Phytophthora infestans (19, 20). However, the potential role of CA in animal or human fungal infections has not yet been elucidated.

CA is also a key anti-inflammatory constituent of several traditional Chinese medicinal formulations and plant extracts, including Kouyanqing Granule, industrial hemp, and Berberis vulgaris L (21–23). Accumulating evidence indicates that CA exerts anti-inflammatory effects in multiple pathological contexts. For example, Kim et al. reported that CA alleviates ulcerative colitis by modulating IL-6 and TNF-α expression (15). CA has also been shown to reduce levels of TNF-α, IL-6, and IL-1β in the hippocampus of depression-model mice, thereby attenuating neuroinflammation (24). Moreover, CA demonstrates therapeutic potential in allergic diseases (14); it suppresses IL-1β expression in nasal mucosa and reduces eosinophil and mast cell infiltration (25). Despite these findings, whether CA confers therapeutic benefits in FK remains unclear.

In the present study, using both *in vitro* and *in vivo* models of *Aspergillus fumigatus* (*AF*) keratitis, we demonstrated that CA exerted direct antifungal activity by inhibiting conidial production and germination, inhibiting mycelial growth, and disrupting fungal cell membranes and cell walls. In addition, CA attenuated corneal inflammation by suppressing neutrophil and macrophage recruitment, reducing pro-inflammatory cytokine production, and increasing anti-inflammatory cytokine production via inhibition of the MyD88/NF-κB signaling pathway. Notably, combined treatment with CA and natamycin produced superior therapeutic outcomes compared with monotherapy. Collectively, our findings identify CA as a promising candidate for the treatment of FK.

## 2 Materials and methods

### 2.1 Preparation of CA

Powder (MedChemExpress, China) was stored at -20 °C for long-term preservation. For experimental use, CA was dissolved in phosphate-buffered saline (PBS) and stored temporarily. Immediately before each experiment, CA was further diluted with PBS or cell culture medium to the desired working concentrations.

### 2.2 Preparation of conidia and mycelium

The *AF* strain used in this study (No. 3.0772) was obtained from the China General Microbiological Culture Collection Center. *AF* was cultured on Sabouraud dextrose agar (SDA) plates at 37 °C for 3-5 days. Fresh conidia were harvested with PBS and stored briefly at 4 °C. For mycelial preparation, *AF* was cultured in Sabouraud liquid medium at 37 °C with shaking at 120 rpm for 3-5 days. The harvested mycelium was homogenized and inactivated by treatment with 75% ethanol overnight. Viable conidia were used for the mouse model of *AF* keratitis and *in vitro* antifungal assays, whereas inactivated mycelium was used for cell stimulation experiments.

### 2.3 Cell model of *AF* keratitis and treatment

RAW264.7 murine macrophage cells were obtained from the Chinese Academy of Sciences and were maintained in high-glucose DMEM supplemented with 10% fetal bovine serum and 1% antibiotic solution. Immortalized human corneal epithelial cells (HCECs) were obtained from the Ocular Surface Laboratory of Xia Men Eye Center and were maintained in α-MEM supplemented with 10% fetal bovine serum, 1% MEM Non-Essential Amino Acid Solution (NEAA) and 1% antibiotic solution.

Prior to stimulation, RAW264.7 cells were pretreated with CA (5 mM), TJ-M2010-5 (40 μM; MedChemExpress), JSH-23 (30 μM; MedChemExpress) or PBS for 1 h, followed by stimulation with inactivated *AF* mycelium. RAW264.7 cells were collected for subsequent analyses at 8 or 16 h after stimulation.

### 2.4 Cell counting kit-8 (CCK-8) assay

Cells were treated with various concentrations of CA or PBS for 24 h. After removal of the culture medium, fresh medium containing CCK-8 solution (Solarbio, China) was added. Cells were incubated for 1 h, and absorbance was measured at 450 nm using a microplate reader, as previously described (26).

### 2.5 Live-dead cell staining

Cells were treated with different concentrations of CA, 5mM H_2_O_2_ or PBS for 24 h. After removal of the medium, cells were incubated with Calcein-AM and propidium iodide (PI) staining solutions (Meilunbio, China) for 30 min. After washing, fluorescent images were acquired using a fluorescence microscope.

### 2.6 Cell scratch assay

Three parallel scratches were created in confluent cell monolayers using a sterile pipette tip. Cells were then cultured in serum-free medium containing various concentrations of CA or PBS for 48 h. Scratch images were captured using an optical microscope at 0, 12, 24, and 48 h after treatment. Scratch widths were measured, and cell migration rates were calculated based on changes in wound width, as previously described (26).

### 2.7 Mice model of *AF* keratitis and treatment

Eight-week-old female C57BL/6 mice were obtained from Jinan Pengyue Laboratory Animal Breeding Co., Ltd. (Shandong, China). All animal procedures were conducted in accordance with the Declaration of Helsinki (Edinburgh revision, 2000) and the Association for Research in Vision and Ophthalmology (ARVO) Statement on the Use of Animals in Ophthalmic and Vision Research. All experimental protocols were reviewed and approved by the Ethics Committee for Laboratory Animal Welfare of Qingdao University (Approval No. 20250916C576J4020251010187).

Mice were randomly assigned to four groups (*n* = 8 per group): PBS group, PBS-treated *AF* keratitis group, CA group, and CA-treated *AF* keratitis group. After intraperitoneal anesthesia with sodium pentobarbital, the right corneal surface was gently scratched using a 30-gauge needle. Subsequently, 2.5 μL of *AF* conidial suspension (5 × 10^7^/mL) was injected into the corneal stroma using a 33-gauge microsyringe (Hamilton, Switzerland). After model induction, mice received daily subconjunctival injections of PBS or CA (5 mM). Corneal changes were examined using slit-lamp microscopy, and clinical scores were recorded as previously described (27).

For combination therapy experiments, mice were divided into four additional groups (*n* = 8 per group): PBS-treated *AF* keratitis group, CA-treated *AF* keratitis group, natamycin-treated *AF* keratitis group, and CA plus natamycin-treated *AF* keratitis group. PBS or CA (5 mM) was administered via daily subconjunctival injection (5 μL). Natamycin eye drops (5%; Senju Pharmaceutical, Japan) were administered topically at a volume of 10 μL, four times daily.

### 2.8 Fluorescein sodium staining of cornea

Mice received daily subconjunctival injections of CA (1 or 5 mM, 5 μL) or PBS. At 0, 1, and 3 days after treatment, corneal epithelial defects were evaluated by fluorescein sodium staining and imaged using a slit-lamp microscope equipped with a cobalt blue filter.

### 2.9 Hematoxylin-eosin (HE) staining

Mouse liver, kidney, and eye tissues were fixed in 4% paraformaldehyde (Solarbio, China) for 3 days, followed by paraffin embedding and sectioning at a thickness of 8 μm. Tissue sections were deparaffinized in xylene, rehydrated through graded ethanol solutions, and stained with hematoxylin and eosin. Histological images were acquired using an optical microscope.

### 2.10 Immunofluorescence staining

Frozen sections (8 μm) of mouse corneal tissue and cell smears were fixed with 4% paraformaldehyde, permeabilized with 0.3% Triton X-100 (Solarbio, China), and blocked with goat serum (BOSTER, China). Samples were then incubated with primary antibodies overnight at 4 °C. After washing, sections were incubated with the appropriate fluorophore-conjugated secondary antibodies for 1 h at room temperature in the dark, followed by nuclear counterstaining with 4′,6-diamidino-2-phenylindole (DAPI; Solarbio, China) for 5 min. Images were acquired using a fluorescence microscope. Primary antibodies used were as follows: anti-NIMP-R14 (Santa Cruz, sc-59338), anti-F4/80 (Cell Signaling Technology, 30325), anti-MyD88 (Proteintech, 67969-1-Ig), and anti-NF-κB p65 (Novus, NB100-56712). Secondary antibodies included Alexa Fluor 488-conjugated anti-rat IgG (CST, 4416S), Alexa Fluor 555-conjugated anti-rabbit IgG (CST, 4413S), and Alexa Fluor 488-conjugated anti-mouse IgG (CST, 4408S).

### 2.11 Myeloperoxidase (MPO) measurement

Mouse corneas were homogenized in lysis buffer using a tissue grinder and centrifuged to collect the supernatant. MPO levels in corneal lysates were quantified using a Mouse MPO Enzyme-Linked Immunosorbent Assay kit (BOSTER, China), according to the manufacturer’s instructions.

### 2.12 Minimum inhibitory concentration (MIC) of *AF*

The MIC of CA against *AF* was determined using the broth microdilution method. *AF* conidia were adjusted to a final inoculum strength of 1-5 × 10^5^ CFU/mL and inoculated into 96-well plates containing Sabouraud liquid medium supplemented with different concentrations of CA (concentration range: 0.1 to 5 mM). The plates were incubated at 37 °C. Fungal growth was assessed by measuring the absorbance at 540 nm using a microplate reader at 0, 12, 24, and 36 h. The MIC was defined as the lowest concentration of CA that resulted in a significant reduction in growth compared to the control.

### 2.13 Calcofluor white staining of *AF*

*AF* conidia were cultured in 6-well plates containing Sabouraud liquid medium with various concentrations of CA at 37 °C for 12 h. The resulting mycelia were stained with calcofluor white (Sigma-Aldrich, USA) for 10 min and imaged using a fluorescence microscope.

### 2.14 PI staining of *AF*

*AF* conidia were cultured in Sabouraud liquid medium in 6-well plates at 37 °C for 12 h, followed by treatment with different concentrations of CA for an additional 12 h. Mycelia were then stained with PI (Meilunbio, China) for 15 min and visualized using a fluorescence microscope.

### 2.15 Corneal fungal burden assay

Mouse corneas were homogenized using a tissue grinder, serially spread onto SDA plates, and incubated at 37 °C. Fungal colony growth was observed and photographed at 18, 24, and 30 h after incubation.

### 2.16 Periodic Acid-Schiff (PAS) staining of cornea

Frozen sections of mouse cornea were stained using a PAS staining kit (Beyotime, China) to visualize *AF* conidia and mycelia. Stained sections were examined and imaged using an optical microscope according to the manufacturer’s protocol.

### 2.17 Quantitative real-time polymerase chain reaction (qRT-PCR)

For the *in vivo* experiments, total RNA was extracted from mouse corneal tissues, while for the *in vitro* experiments, total RNA was extracted from RAW264.7 cells and *AF*. Total RNA was extracted using the RNAiso Plus kit (Takara, China). After assessment of RNA concentration and purity, reverse transcription was performed using HiScript III RT SuperMix for qPCR (Vazyme, China). Quantitative PCR was carried out using ChamQ Universal SYBR qPCR Master Mix (Vazyme, China). Relative gene expression levels were calculated using the 2^−ΔΔCt^ method, with β-actin or 18S rRNA serving as the internal control. Primer sequences used in this study are listed in **Table 1**.

**Table 1:** Nucleotide sequences of primers for quantitative RT-PCR.

Gene	Primer sequence (5’-3’)
Mouse IL-1β Mouse IL-6 Mouse TNF-α Mouse IL-10 Mouse β-actin *A. fumigatus HSP90* *A. fumigatus BrlA* *A. fumigatus AbaA* *A. fumigatus WetA* *A. fumigatus CnaA* *A. fumigatus CrzA* *A. fumigatus 18S rRNA*	F-CGCAGCAGCACATCAACAAGAGC; R-TGTCCTCATCCTGGAAGGTCCACG F-CACAAGTCCGGAGAGGAGAC; R-CAGAATTGCCATTGCACAAC F-ACCCTACACTCAGATCATCTT; R-GGTTGTCTTTGAGATCCATGC F-TGCTAACCGACTCCTTAATGCAGGAC; R-CCTTGATTTCTGGGCCATGCTTCTC F-CATCCGTAAAGACCTCTATGCCAAC; R-ATGGAGCCACCGATCCACA F-CTCCCTTCCTTGACAGCCTCA; R-GTCCACCAGCTTCTTGCCATC F-CGCAGTGACAACCTCAATGCC; R-TCGGGACTCGTCTCATCCAAG F-TGAGGACGCTTTTCAGCAAGC; R-CGTTTCGGCCATACGACTTTCC F-CCGAGTGGGAGTTCCAAGACT; R-AGCCTCCAGTGCTTCCACATC F-GATCCGTCCGAGACGAGGTATC; R-GATGCCTGCATTCGTGGTTGC F-ATATGCCCATCCCAGCACCAG; R-CGGAGAAAGTGAGCGAGGAGT F-CTTAAATAGCCCGGTCCGCATT; R-CATCACAGACCTGTTATTGCCG

* F, forward; R, reverse.

### 2.18 Western blot analysis

Cells or tissues were lysed in high-efficiency RIPA buffer (Solarbio, China), and protein concentrations were determined using a BCA protein assay kit (Solarbio, China). Equal amounts of protein were separated by sodium dodecyl sulfate polyacrylamide gel electrophoresis (SDS-PAGE) and transferred onto polyvinylidene difluoride (PVDF) membranes (Merck, Germany). After blocking for 2 h, membranes were incubated with primary antibodies overnight at 4 °C, followed by incubation with the appropriate secondary antibodies for 1 h at room temperature. Protein bands were visualized using enhanced chemiluminescence reagents, and band intensities were quantified using ImageJ software.

Primary antibodies included anti-IL-1β (R&D Systems, AF-401-NA), anti-IL-6 (Proteintech, 66146-1-Ig), anti-TNF-α (Proteintech, 60291-1-Ig), anti-IL-10 (Proteintech, 20850-1-AP), anti-MyD88 (Proteintech, 67969-1-Ig), anti-NF-κB p65 (Novus, NB100-56712), anti-phospho-NF-κB p65 (Wanleibio, WL02169), and anti-β-actin (Elabscience, E-AB-20058). Secondary antibodies included HRP-conjugated anti-mouse IgG (Abclonal, AS003), anti-goat IgG (Abclonal, AS029), and anti-rabbit IgG (Abclonal, AS014).

### 2.19 Transwell migration assay

5 × 10^4^ RAW264.7 cells in DMEM serum-free medium with CA (5 mM) or PBS, were seeded into the upper chamber of a Transwell insert (8 μm pore size; LABSELECT). α-MEM with 10% fetal bovine serum and 1% NEAA, and inactivated *AF* mycelium were added in the lower chamber. After incubation for 24 h, the Transwell chamber was taken out and washed with PBS. The cells were fixed with 4% paraformaldehyde for 20 min and stained with 0.1% crystal violet for 30 min. The upper unmigrated cells were gently wiped off with a moistened cotton swab. Images were acquired using an optical microscope.

### 2.20 Statistical analysis

Data were presented as mean ± standard deviation (SD) from at least three independent experiments. Statistical analyses were performed using GraphPad Prism software (version 10.0). Comparisons between two groups were conducted using an unpaired two-tailed Student’s t test. Comparisons among multiple groups were analyzed using one-way analysis of variance (ANOVA). A *P* value of <0.05 was considered statistically significant.

## 3 Results

### 3.1 *In vitro* and *in vivo* safety evaluation of CA

**Figure 1:**
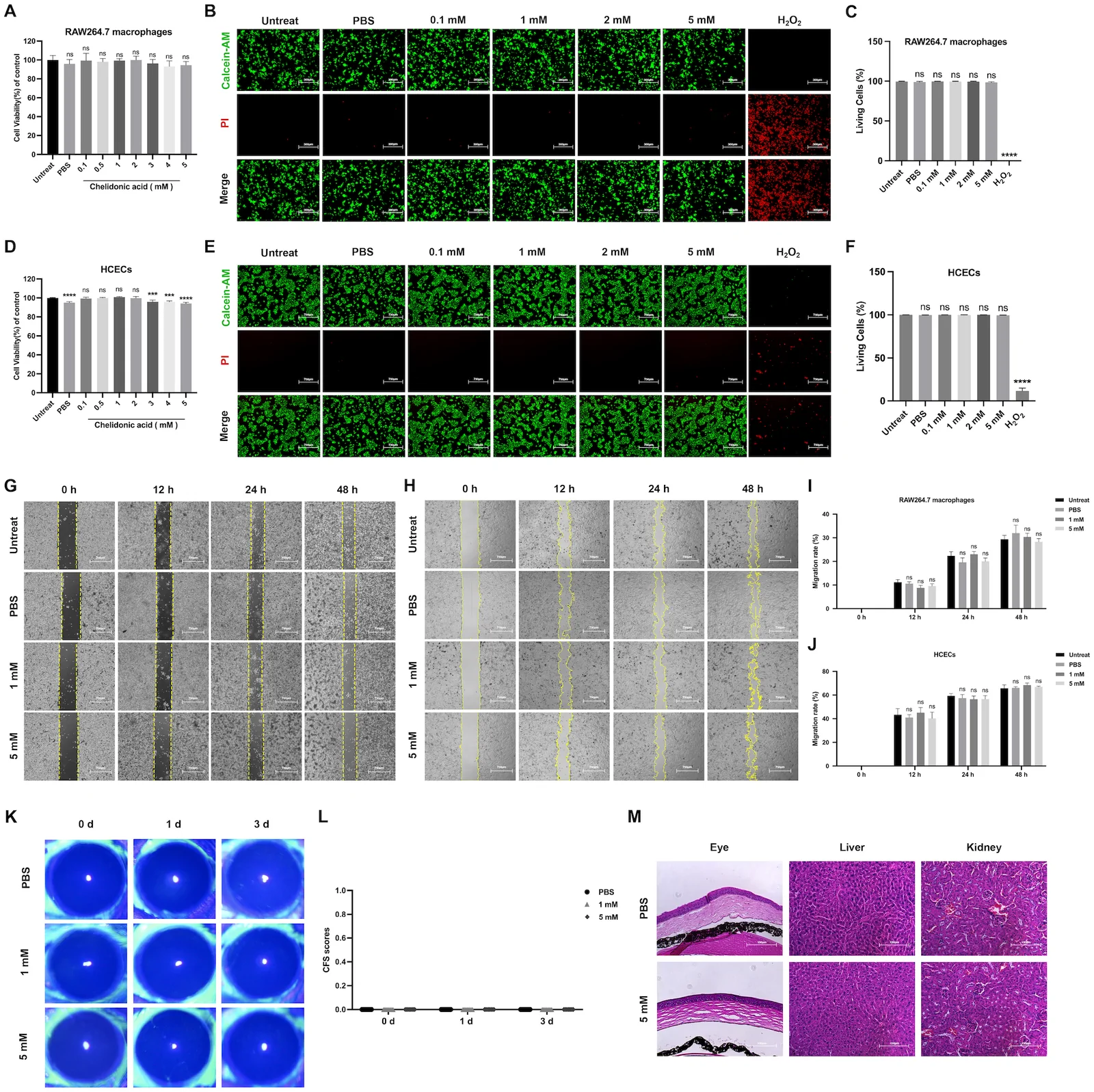
Safety analysis of CA in RAW264.7 cells, HCECs and mice. **(A)** Cell viability detected using CCK-8 after treating RAW264.7 cells with 0.1; 0.5; 1; 2; 3; 4; or 5 mM CA or PBS for 24 hours; **(B)** Live-dead cell staining and **(C)** living cells rate of RAW264.7 cells treated with 0.1; 1; 2; or 5 mM CA, 5mM H_2_O_2_ or PBS for 24 hours. Magnification ×100; **(D)** Cell viability detected using CCK-8 after treating HCECs with 0.1; 0.5; 1; 2; 3; 4; or 5 mM CA or PBS for 24 hours; **(E)** Live-dead cell staining and **(F)** living cells rate of HCECs treated with 0.1; 1; 2; or 5 mM CA, 5mM H_2_O_2_ or PBS for 24 hours. Magnification ×40; **(G, I)** Migration rate of RAW264.7 cells treated with 1 or 5 mM CA or PBS at 0; 12; 24; and 48 hours. Magnification ×40; **(H, J)** Migration rate of HCECs treated with 1 or 5 mM CA or PBS at 0; 12; 24; and 48 hours. Magnification ×40; **(K)** Fluorescein sodium staining and **(L)** CFS (corneal fluorescein sodium) score of mouse corneas treated with 1 or 5 mM CA or PBS at 0; 1; and 3 days (*n* = 8 mice/group); **(M)** HE staining of eye, liver and kidney tissues from mice treated with 5 mM CA or PBS for 3 days. Magnification ×200. ns, not significant; ***P < 0.001; ****P < 0.0001; CA, chelidonic acid; PBS, phosphate-buffered saline; HE, hematoxylin-eosin. Data are presented as mean ± SD from three independent experiments.

To determine the safety profile and optimal working concentration of CA, a series of *in vitro* and *in vivo* assays was performed. CCK-8 analysis showed that treatment of RAW264.7 cells or HCECs with CA at concentrations ranging from 0.1 to 5 mM for 24 h did not significantly affect cell viability, which remained above 90% at all tested doses (**Figures 1A, D**). Consistently, live-dead staining revealed no significant increase in the number of dead RAW264.7 cells or HCECs in CA-treated groups compared with the control (**Figures 1B, C, E, F**). In contrast, treatment with 5 mM H_2_O_2_, which served as a positive cytotoxic control, resulted in a marked increase in PI-positive cells, confirming the sensitivity and reliability of the staining system. Cell scratch assays further demonstrated that exposure to 1 or 5 mM CA did not impair the migratory capacity of RAW264.7 cells or HCECs (**Figures 1G, H, I, J**). *In vivo*, fluorescein sodium staining showed no evidence of corneal epithelial damage in mice receiving subconjunctival injections of CA at 1 or 5 mM for 1 or 3 consecutive days (**Figures 1K, L**). Moreover, HE staining of eye, liver and kidney tissues revealed no histopathological abnormalities following CA treatment (**Figure 1M**). Collectively, these results indicate that CA is well tolerated *in vitro* and *in vivo*, and that 5 mM represents a safe concentration for subsequent experiments.

### 3.2 Antifungal activity of CA against *AF*

**Figure 2:**
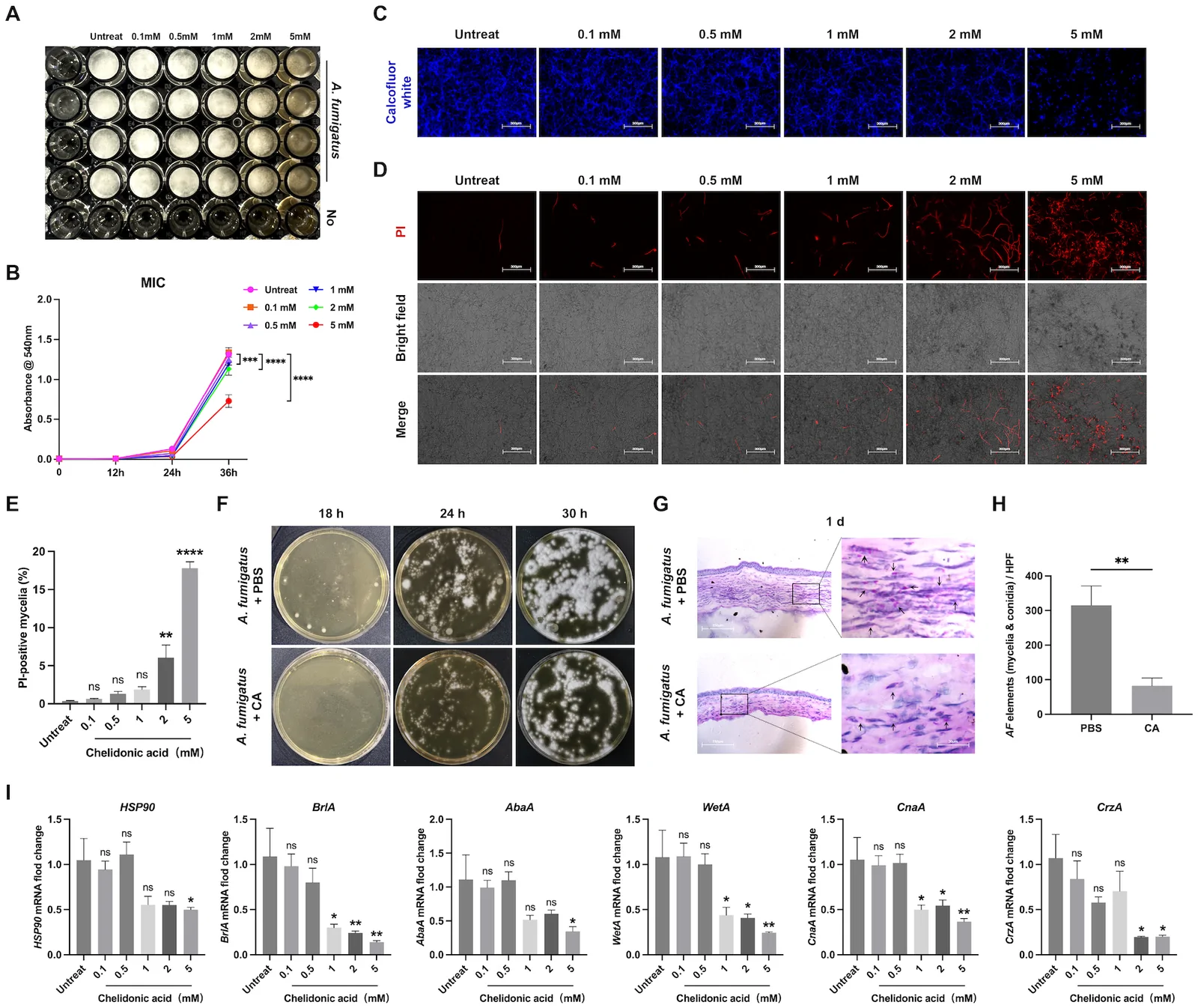
The antifungal activity of CA. **(A)** Mycelial growth of *AF* after 36 hours of cultivation in media containing 0.1; 0.5; 1; 2; or 5 mM CA; **(B)** MIC of CA against *AF*; **(C)** Calcofluor white staining and **(D, E)** PI staining of *AF* treated with 0.1; 0.5; 1; 2; or 5 mM CA. Magnification ×100; **(F)** Fungal colonies cultured for 18; 24; and 30 hours from corneas of mice with *AF* keratitis treated with 5 mM CA or PBS; **(G, H)** PAS staining of corneal sections and quantification. The number of PAS-positive *AF* elements (including mycelium and conidia) was counted and expressed as the average number per HPF. Magnification ×200 and Magnification ×1000; **(I)** mRNA expression levels of *HSP90*, *BrlA*, *AbaA*, *WetA*, *CnaA*, and *CrzA* in *AF* detected by qRT-PCR. *n* = 8 mice/group. *P < 0.05; **P < 0.01; ***P < 0.001; ****P < 0.0001; ns, not significant; *AF*, *Aspergillus fumigatus*; CA, chelidonic acid; PBS, phosphate-buffered saline; MIC, Minimum inhibitory concentration; PI, propidium iodide; HPF, high-power field. Data are presented as mean ± SD from three independent experiments.

To assess the antifungal effects of CA, both *in vitro* and *in vivo* antifungal assays were performed. At 36 h post-inoculation, mycelial growth was markedly suppressed in the 5 mM CA group compared with the control (**Figure 2A**), with a corresponding decrease in absorbance at 540 nm (**Figure 2B**). Mechanistic studies using calcofluor white staining revealed fewer mycelia and a higher proportion of ungerminated conidia in the CA-treated group (**Figure 2C**), indicating inhibition of conidial germination and mycelial growth. PI staining showed increased numbers of PI-positive mycelia in the 2 mM CA and 5 mM CA group (**Figures 2D, E**), suggesting that CA disrupts fungal cell membrane integrity. Consistent with these findings, CA treatment reduced corneal fungal loads in *AF* keratitis mice compared with untreated controls (**Figure 2F**). PAS staining further confirmed diminished mycelial and conidial presence in CA-treated corneas (**Figures 2G, H**). To investigate the mechanism of antifungal activity, we examined the *HSP90* and calcineurin pathway. qRT-PCR results showed that the expression of *HSP90*, *BrlA*, *AbaA*, *WetA*, *CnaA* and *CrzA* in *AF* from the 1, 2, and 5 mM CA treatment groups was lower than that in the control group, with the 5 mM CA treatment exhibiting the strongest inhibitory effect (**Figure 2I**). Collectively, these results demonstrate that CA possesses antifungal activity against *AF*.

### 3.3 CA attenuated *AF* keratitis by inhibiting the recruitment of neutrophils and macrophages

**Figure 3:**
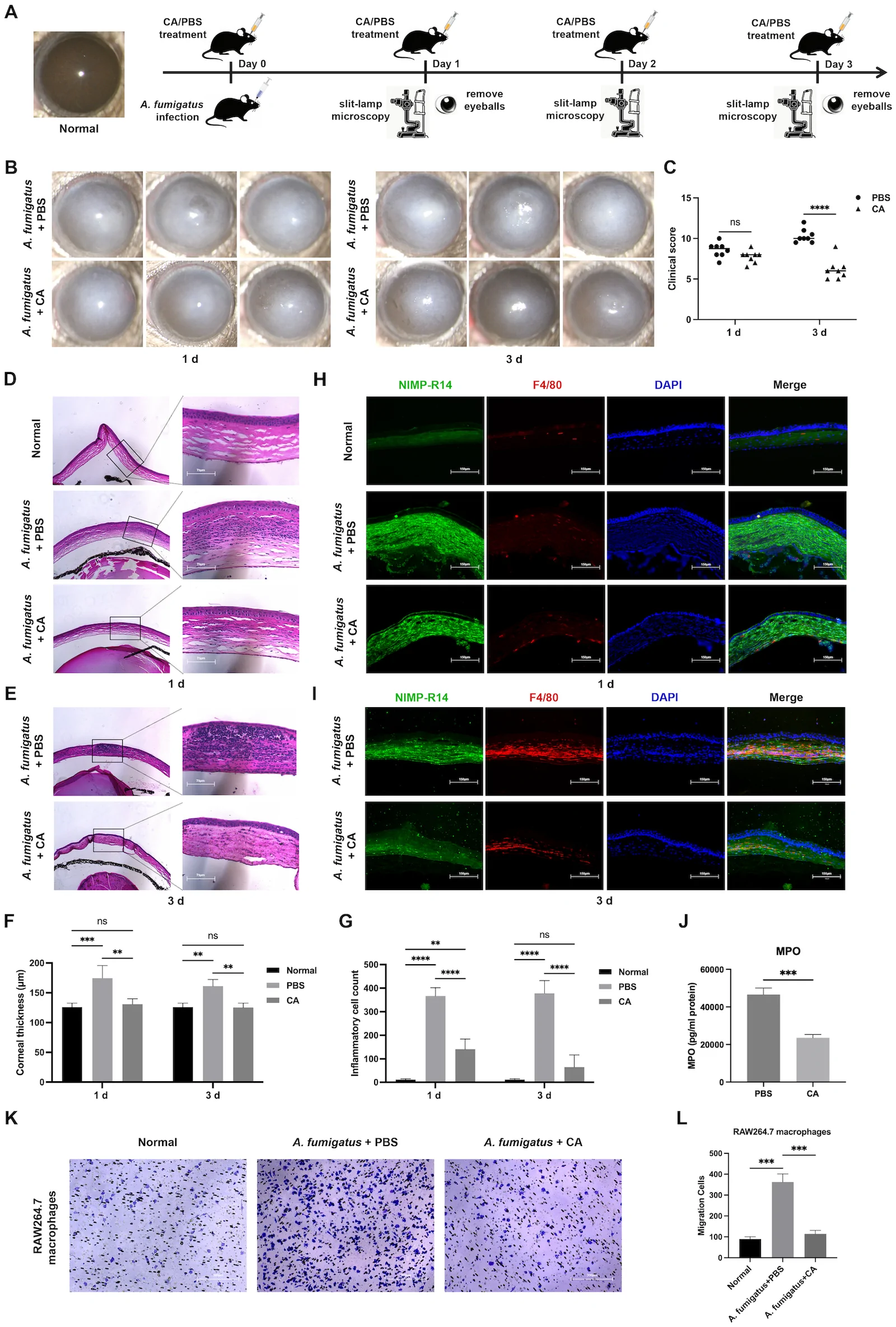
CA alleviates the severity of *AF* keratitis by inhibiting the recruitment of neutrophils and macrophages. **(A)** Normal cornea; and time course for *AF* keratitis mouse modeling; CA or PBS treatment; and eye enucleation; **(B)** Slit-lamp microscopy images and **(C)** clinical scores of mice with *AF* keratitis at 1 and 3 days post-infection; **(D, E)** Corneal HE staining; **(F)** corneal thickness; and **(G)** inflammatory cell counts in mice with *AF* keratitis at 1 and 3 days post-infection. Magnification ×400; **(H, I)** Immunofluorescence staining of corneal neutrophils and macrophages in *AF* keratitis mice at 1 and 3 days post-infection. Magnification ×200; **(J)** Corneal MPO levels in *AF* keratitis mice at 3 days post-infection; **(K, L)** Transwell migration assay of RAW264.7 cells treated with CA or PBS towards *AF.* Magnification ×100. *n* = 8 mice/group. **P < 0.01; ***P < 0.001; ****P < 0.0001; ns, not significant; *AF*, *Aspergillus fumigatus*; CA, chelidonic acid; PBS, phosphate-buffered saline; MPO, myeloperoxidase. Data are presented as mean ± SD from three independent experiments.

The aforementioned studies have confirmed that 5 mM CA is safe and exhibits good antifungal activity in both *in vitro* and *in vivo* assays. Therefore, we have selected 5 mM CA as the drug concentration for subsequent experiments. A murine model of *AF* keratitis was established to evaluate the therapeutic efficacy of CA (**Figure 3A**). Slit-lamp examination and corneal HE staining revealed that *AF* infection induced pronounced corneal opacity (**Figure 3B**), extensive epithelial ulceration (**Figure 3B**), increased corneal thickness (**Figures 3D–F**), and marked inflammatory cell infiltration (**Figures 3D, E, G**). In contrast, CA-treated mice exhibited substantially reduced corneal opacity and ulcer area (**Figure 3B**), lower clinical scores (**Figure 3C**), decreased corneal thickness (**Figures 3D–F**), and attenuated inflammatory cell infiltration (**Figures 3D, E, G**) at both 1 and 3 days post-infection. To further assess the effects of CA on inflammatory cell recruitment, immunofluorescence staining was performed to detect neutrophils and macrophages in infected corneas. At day 1 post-infection, extensive neutrophil infiltration was observed in the corneal stroma of control mice, whereas CA treatment markedly reduced neutrophil accumulation (**Figure 3H**). At this time point, macrophage infiltration was minimal in both groups (**Figure 3H**). By day 3 post-infection, infiltration of both neutrophils and macrophages was significantly decreased in CA-treated corneas compared with controls (**Figure 3I**). Consistently, MPO activity, an indicator of neutrophil accumulation, was significantly reduced in the CA-treated group at day 3 (**Figure 3J**). Transwell migration assay showed that CA reduced the migration of RAW264.7 cells towards *AF* (**Figures 3K, L**). Together, these findings demonstrated that CA alleviated the severity of *AF* keratitis, as evidenced by improved clinical outcomes and reduced inflammatory cell infiltration, primarily through suppression of neutrophil and macrophage recruitment.

### 3.4 CA suppressed pro-inflammatory cytokine expression and promoted anti-inflammatory cytokine expression in *AF* keratitis

**Figure 4:**
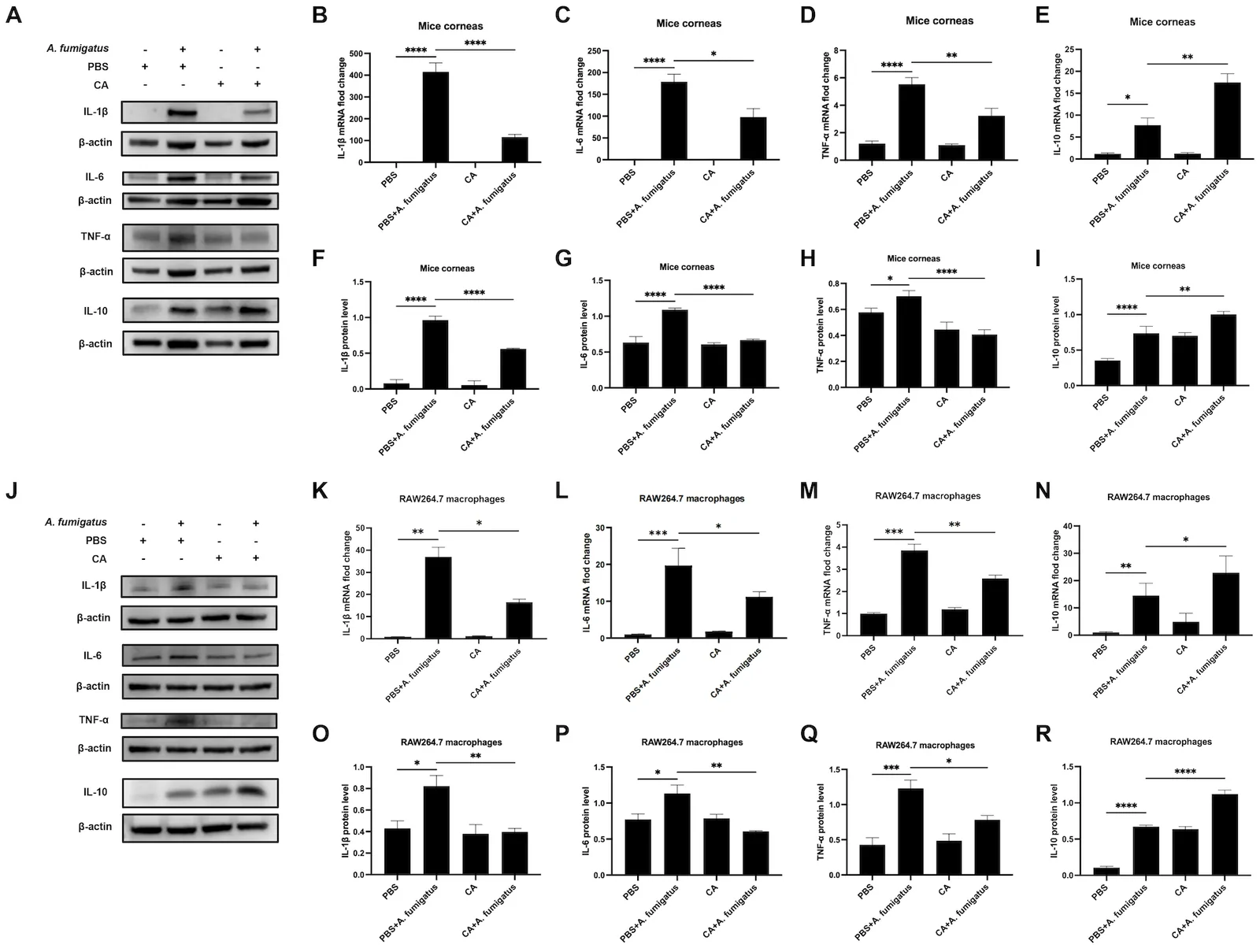
CA inhibits the expression of pro-inflammatory cytokine and promotes the expression of anti-inflammatory cytokine in *AF* keratitis. mRNA expression levels of IL-1β **(B)**; IL-6 **(C)**; TNF-α **(D)**; and IL-10 **(E)** in corneas of *AF* keratitis mice detected by qRT-PCR; Protein expression levels of IL-1β **(A, F)**; IL-6 **(A, G)**; TNF-α **(A, H)**; and IL-10 **(A, I)** in corneas of *AF* keratitis mice detected by Western blot; mRNA expression levels of IL-1β **(K)**; IL-6 **(L)**; TNF-α **(M)**; and IL-10 **(N)** in *AF*-stimulated RAW264.7 cells detected by qRT-PCR; Protein expression levels of IL-1β **(J, O)**; IL-6 **(J, P)**; TNF-α **(J, Q)**; and IL-10 **(J, R)** in *AF*-stimulated RAW264.7 cells detected by Western blot. *n* = 8 mice/group. *P < 0.05; **P < 0.01; ***P < 0.001; ****P < 0.0001; *AF*, *Aspergillus fumigatus*; CA, chelidonic acid; IL, interleukin; TNF-α, tumor necrosis factor-alpha; PBS, phosphate-buffered saline. Data are presented as mean ± SD from three independent experiments.

To assess the effects of CA in inflammation, the expression of key pro-inflammatory cytokines and anti-inflammatory cytokine were evaluated. The excessive expression of pro-inflammatory cytokines, such as IL-1β, IL-6, and TNF-α, can intensify the inflammatory response and cause corneal damage (9, 10). On the contrary, the anti-inflammatory cytokine IL-10 can inhibit excessive pro-inflammatory responses and promote the resolution of inflammation, thereby reducing corneal damage. qRT-PCR and western blot analyses revealed that CA treatment significantly decreased the expression of pro-inflammatory cytokines IL-1β, IL-6, and TNF-α at both mRNA and protein levels in the corneas of *AF*-infected mice (**Figures 4A–D, F-H**), while simultaneously increasing the expression of anti-inflammatory cytokine IL-10 at both mRNA and protein levels (**Figures 4A, E, I**). Similarly, RAW264.7 cells stimulated with *AF* exhibited upregulated IL-1β, IL-6, and TNF-α expression, whereas treatment with CA effectively attenuated these pro-inflammatory cytokines expression (**Figures 4J–M, O-Q**). The treatment with CA also promoted the expression of the anti-inflammatory cytokine IL-10 in RAW264.7 cells stimulated by *AF* (**Figures 4J, N, R**). These results indicate that CA suppressed pro-inflammatory cytokine expression and promoted anti-inflammatory cytokine expression in *AF* keratitis, which is beneficial for alleviating the inflammatory response and corneal damage.

### 3.5 CA inhibited the MyD88/NF-κB signaling pathway in *AF* keratitis

**Figure 5:**
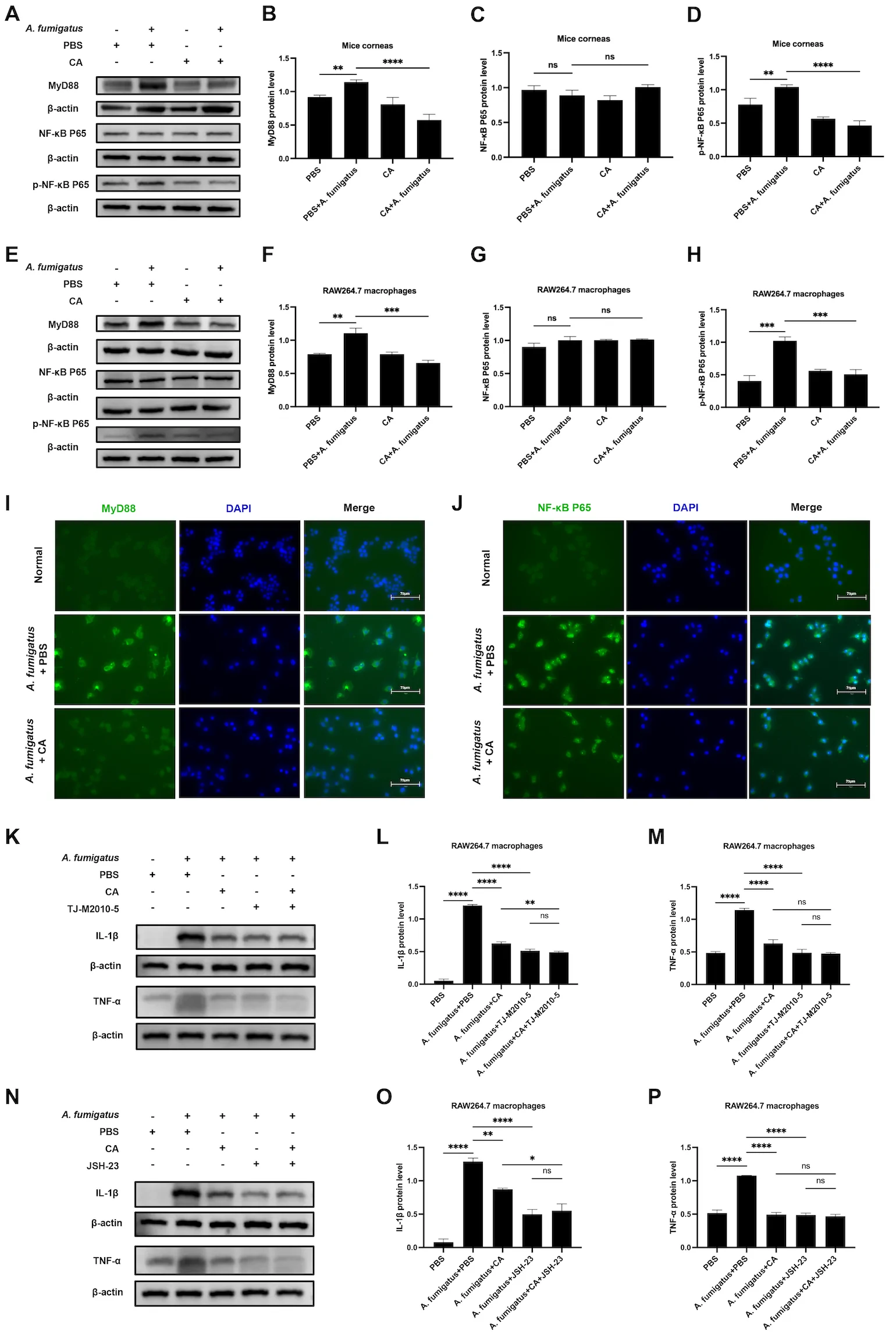
CA inhibits the MyD88/NF-κB signaling pathway in *AF* keratitis. Protein expression levels of MyD88 **(A, B)**; NF-κB p65 **(A, C)**; and p-NF-κB p65 **(A, D)** in corneas of *AF* keratitis mice detected by Western blot; Protein expression levels of MyD88 **(E, F)**; NF-κB p65 **(E, G)**; and p-NF-κB p65 **(E, H)** in *AF*-stimulated RAW264.7 cells detected by Western blot; **(I, J)** Expression and localization of MyD88 and NF-κB p65 in *AF*-stimulated RAW264.7 cells detected by immunofluorescence staining. Magnification ×400; After TJ-M2010–5 pre-treatment, the protein expression levels of IL-1β **(K, L)** and TNF-α **(K, M)** in *AF*-stimulated RAW264.7 cells detected by Western blot; After JSH-23 pre-treatment, the protein expression levels of IL-1β **(N, O)** and TNF-α **(N, P)** in *AF*-stimulated RAW264.7 cells detected by Western blot. *n* = 8 mice/group. *P < 0.05; **P < 0.01; ***P < 0.001; ****P < 0.0001; ns, not significant; *AF*, *Aspergillus fumigatus*; CA, chelidonic acid; MyD88, myeloid differentiation factor 88; NF-κB, nuclear factor-kappa B; TJ-M2010-5, MyD88 inhibitor; JSH-23, NF-κB inhibitor. Data are presented as mean ± SD from three independent experiments.

To elucidate the mechanism underlying the anti-inflammatory effects of CA, we examined the MyD88/NF-κB pathway, a canonical inflammatory cascade that drives pro-inflammatory cytokine transcription upon fungal recognition (28, 29). Given that CA suppressed the expression of these cytokines, we hypothesized that CA may act through this signaling axis. Western blot analysis showed that *AF* infection elevated protein levels of MyD88 in both corneal tissue and RAW264.7 cells, whereas CA treatment significantly reduced these levels (**Figures 5A, B, E, F**). Total NF-κB p65 protein levels remained unchanged across all groups (**Figures 5A, C, E, G**); however, CA treatment reduced the expression levels of phosphorylated NF-κB p65 in corneal tissue and RAW264.7 cells following *AF* infection (**Figures 5A, D, E, H**). Immunofluorescence staining revealed that CA decreased MyD88 fluorescence and reduced nuclear translocation of NF-κB p65 in *AF*-stimulated RAW264.7 cells (**Figures 5I, J**). To validate whether the anti-inflammatory effect of CA mediated by the MyD88/NF-κB axis, RAW264.7 cells were pretreated with TJ-M2010-5 (a specific MyD88 inhibitor) or JSH-23 (a specific NF-κB inhibitor), followed by stimulation with *AF* in the presence or absence of CA. Western blot analyses showed that CA treatment, TJ-M2010–5 treatment, and JSH-23 treatment each significantly reduced IL-1β and TNF-α expression in *AF*-stimulated RAW264.7 cells. However, the combination of CA with TJ-M2010–5 or JSH-23 did not produce additional suppression beyond that achieved by the inhibitors alone (**Figures 5K–P**). These results demonstrate that CA and the MyD88/NF-κB inhibitors act through the same signaling axis, providing strong evidence that CA’s anti-inflammatory effect is indeed dependent on the inhibition of the MyD88/NF-κB pathway.

### 3.6 Enhanced therapeutic efficacy of CA combined with natamycin in *AF* keratitis

**Figure 6:**
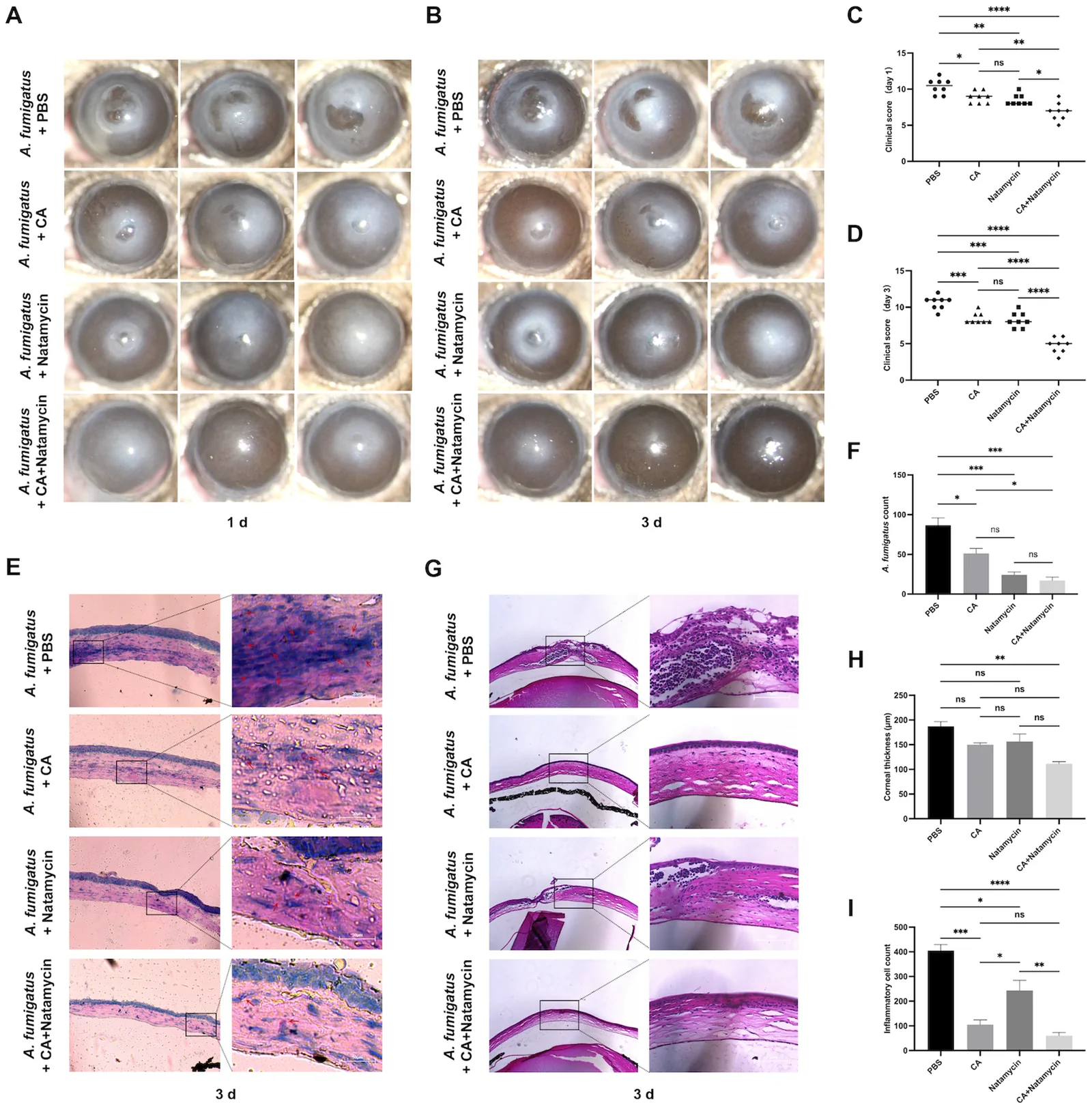
Therapeutic effect of CA and natamycin combination in *AF* keratitis. Slit-lamp microscopy images **(A, B)** and clinical scores **(C, D)** of mice with *AF* keratitis treated with PBS; CA; natamycin; or CA combined with natamycin on days 1 and 3; **(E)** Corneal PAS staining and **(F)** *AF* count of mice treated with PBS; CA; natamycin; or CA combined with natamycin at day 3. Magnification ×1000; **(G)** Corneal HE staining, **(H)** corneal thickness, and **(I)** inflammatory cell counts of mice treated with PBS; CA; natamycin; or CA combined with natamycin at day 3. Magnification ×400. *n* = 8 mice/group. *P < 0.05; **P < 0.01; ***P < 0.001; ****P < 0.0001; ns, not significant; *AF*, *Aspergillus fumigatus*; CA, chelidonic acid; PBS, phosphate-buffered saline; HE, hematoxylin-eosin; PAS, periodic acid-Schiff. Data are presented as mean ± SD from three independent experiments.

To achieve superior therapeutic outcomes, CA was administered in combination with natamycin in the *AF* keratitis mouse model. Slit-lamp imaging revealed that both CA- and natamycin-treated groups exhibited reduced corneal opacity and ulcer area compared with the untreated control, accompanied by decreased clinical scores at both 1 and 3 days post-infection (**Figures 6A–D**). Notably, the CA plus natamycin group showed superior therapeutic outcomes, with further reductions in corneal opacity, ulceration, and clinical scores relative to either monotherapy (**Figures 6A–D**). PAS staining confirmed that combination therapy markedly decreased the presence of mycelia and conidia in the corneal tissue (**Figures 6E, F**). Consistently, HE staining demonstrated that co-treatment with CA and natamycin effectively reduced corneal thickness and inflammatory cell infiltration (**Figures 6G–I**). These results indicate that combining CA with natamycin produces enhanced therapeutic effects in *AF* keratitis.

## 4 Discussion

FK is an infectious disorder of the cornea, primarily caused by pathogenic fungal invasion. Due to subtle early-stage symptoms, patients often present with advanced disease at the time of consultation. Treatment typically involves topical or systemic administration of antifungal agents, such as natamycin, in combination with the host immune response to eradicate the pathogen (30, 31). While an appropriate inflammatory response aids fungal clearance, excessive inflammation can exacerbate corneal ulceration, and in severe cases, lead to corneal perforation or even vision loss (26). These considerations highlight the urgent need for novel therapeutic agents that possess both antifungal and anti-inflammatory properties.

CA, a glutamate decarboxylase inhibitor (32), exhibits diverse pharmacological activities, including anti-inflammatory, anti-allergic, osteogenic, and anticancer effects (14, 15, 17). In the present study, we explored the therapeutic potential of CA in *AF* keratitis.

Safety evaluations conducted *in vitro* and *in vivo* confirmed that 5 mM CA did not compromise the viability or migratory capacity of RAW264.7 cells and HCECs, as demonstrated by CCK-8 assays, live-dead staining, and scratch assays. Similarly, fluorescein sodium staining of mouse corneas and HE staining of eye, liver and kidney tissues revealed no signs of toxicity. These findings establish that 5 mM CA is safe for both cellular and animal models, consistent with prior reports demonstrating CA’s cardioprotective effects in rat models of doxorubicin-induced myocardial injury (33, 34).

**Figure 7:**
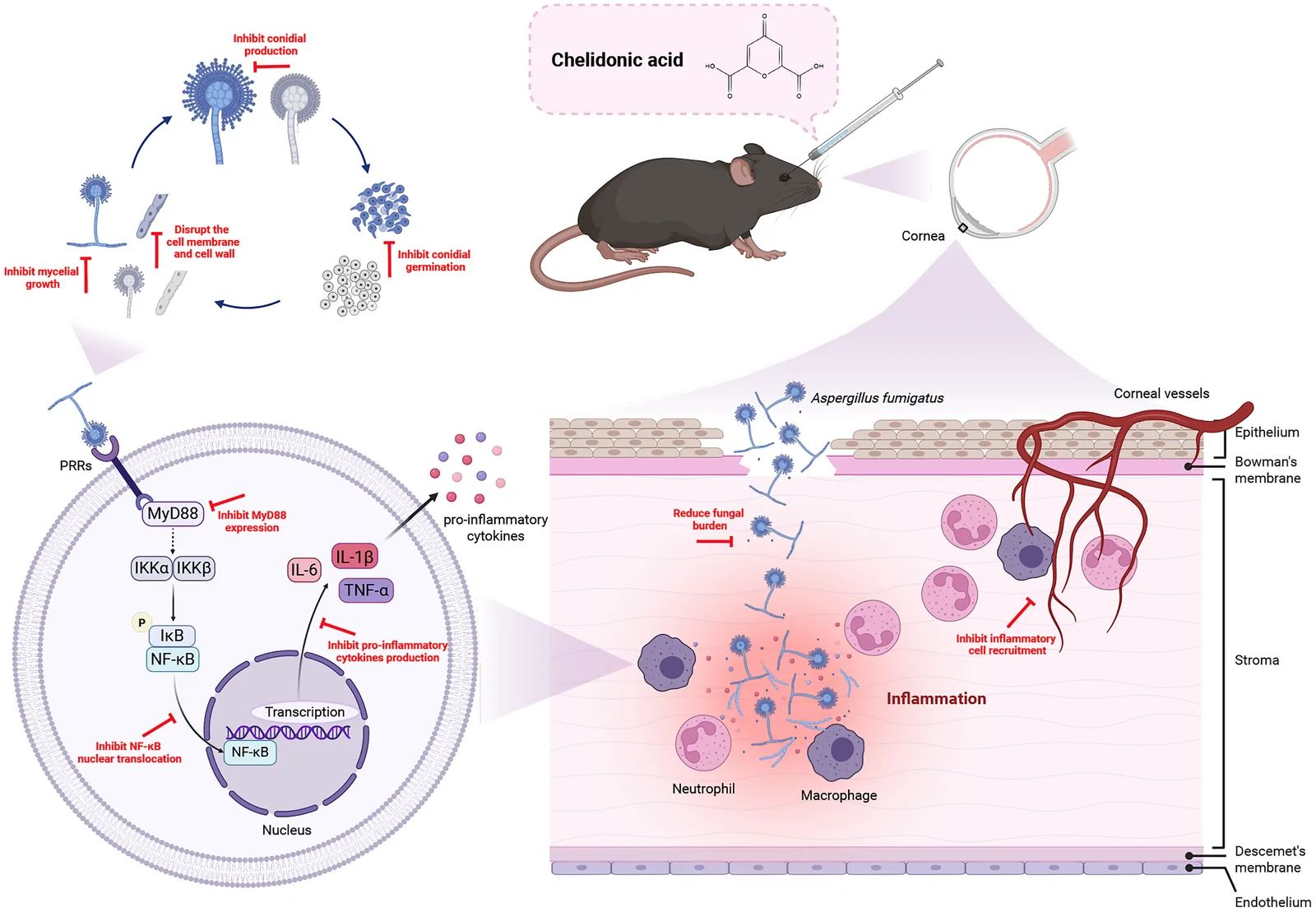
CA exhibits both antifungal and anti-inflammatory activities in AF keratitis. CA exerts antifungal activity by inhibiting conidial production and germination, inhibiting mycelial growth, and disrupting fungal cell membranes and cell walls. CA exerts anti-inflammatory activity by suppressing neutrophil and macrophage recruitment, and reducing pro-inflammatory cytokines production via inhibition of the MyD88/NF-κB signaling pathway. Created in BioRender. Wenting, L. (2026) https://BioRender.com/x9tdzds.

Evaluation of antifungal activity is a critical component in the development of therapeutic agents for FK. While CA has been reported to inhibit fungal growth in plants (19, 20), its efficacy in animal models of fungal infection had not been investigated. In this study, 5 mM CA inhibited conidial germination and mycelial growth of *AF*, disrupted mycelial cell membranes, and induced fungal cell death. *In vivo*, CA treatment significantly reduced the presence of conidia and mycelia in the corneas of *AF*-infected mice (**Figure 7**). *HSP90*, a molecular chaperone, is essential for conidiation and cell wall integrity in *AF* (35). It participates in conidiation by regulating the expression of the *BrlA*, *AbaA*, and *WetA* genes (36). Calcineurin plays multiple regulatory roles in fungi, including cell wall integrity, drug resistance, growth, and virulence. *CnaA* and *CrzA* are key targets for inhibiting calcineurin in fungi (37). Our findings confirm that CA inhibits the expression of *HSP90*, *BrlA*, *AbaA*, *WetA*, *CnaA*, and *CrzA* in *AF*, suggesting that CA may affect conidial production in *AF*, disrupt its cell wall, and reduce its virulence. These findings demonstrate that CA possesses direct antifungal activity in *AF* keratitis and extend its potential application from plant to mammalian fungal infections.

Neutrophils and macrophages are pivotal during the early stages of FK. Upon fungal infection, host cells initiate a cascade of responses, releasing chemokines and pro-inflammatory cytokines that recruit large numbers of neutrophils and macrophages (38). While these immune cells contribute to fungal clearance via reactive oxygen species and antimicrobial granules, the concomitant release of proteases can damage the corneal stroma, resulting in opacity and scar formation (39, 40). Therefore, limiting excessive activation and recruitment of neutrophils and macrophages is critical to preventing corneal injury. In our study, mice with *AF* keratitis were treated with 5 mM CA, and disease severity was assessed using a clinical scoring system based on ulcer area, opacity, and ulcer morphology (27). CA treatment significantly reduced disease severity, as evidenced by improved corneal transparency, decreased ulcer area, and lower clinical scores. HE staining of corneas revealed reduced corneal thickness and diminished inflammatory cell infiltration following CA administration. Immunofluorescence analysis further demonstrated that CA suppressed neutrophil recruitment on day 1 post-infection and inhibited both neutrophil and macrophage infiltration by day 3. Consistently, MPO levels were reduced in CA-treated corneas, indicating attenuated neutrophil activation and infiltration. The Transwell assay further confirmed that CA can inhibit the migration of macrophages towards *AF*. Collectively, these results indicate that CA mitigates tissue-destructive inflammatory responses in *AF* keratitis by modulating excessive neutrophil and macrophage recruitment (**Figure 7**).

Previous studies have demonstrated that CA reduces the expression of inflammation-related genes by inhibiting NF-κB activation and nuclear translocation, thereby ameliorating AD-like skin lesions (41). In FK, PRRs on corneal host cells detect fungal PAMPs, leading to recruitment of MyD88 (28, 42). Activation of the MyD88 signaling cascade promotes phosphorylation and nuclear translocation of NF-κB p65, which in turn drives transcriptional upregulation of pro-inflammatory cytokines (29, 43). Consistent with these mechanisms, our study demonstrates that CA exerts anti-inflammatory effect by inhibiting MyD88 expression, inhibiting NF-κB p65 phosphorylation and nuclear translocation, suppressing upregulation of pro-inflammatory cytokines IL-1β, IL-6 and TNF-α, and promoting upregulation of anti-inflammatory cytokine IL-10 (**Figure 7**). These findings align with previous reports showing that CA reduced IL-6 production via NF-κB regulation and mitigated renal and neuronal injury by lowering IL-1β, IL-6, and TNF-α levels (44–46).

Natamycin is the first-line therapy for FK; however, it is limited by poor corneal permeability, low bioavailability, and the potential for resistance (47, 48). Moreover, natamycin possesses only antifungal activity and lacks anti-inflammatory properties, rendering it insufficient to prevent corneal damage caused by excessive inflammation (49). In the present study, combined treatment with CA and natamycin more effectively reduced corneal opacity, edema, and ulcer area, lowered clinical scores, and decreased both inflammatory cell infiltration and fungal burden in the cornea. These findings indicate that combining CA with natamycin produces enhanced therapeutic effects, suggesting a promising strategy for *AF* keratitis.

Several natural small molecules have been reported to possess antifungal activity against FK, including curcumin, gallic acid, and others (50, 51). Compared with these compounds, CA offers several unique advantages. First, unlike many natural products that possess either antifungal or anti-inflammatory activity alone, CA exhibits both properties simultaneously. Second, CA demonstrated a favorable safety profile in both cellular and animal models at therapeutic concentrations, with no detectable ocular or systemic toxicity. Third, combined treatment with CA and natamycin produced superior therapeutic outcomes. Collectively, these distinguishing features position CA as a promising lead compound for further preclinical development as a novel therapeutic agent for FK. Nevertheless, limitations could not be avoided. First, while the downregulation of *HSP90* and calcineurin pathway components following CA treatment, the present study does not identify the primary molecular target and dominant downstream effector pathway of CA in *AF*. In the future, it is necessary to determine the direct molecular targets of CA in *AF* through biochemical and genetic methods. Second, while subconjunctival injection was chosen for this study to ensure local bioavailability, future translational studies should focus on developing novel formulations to enhance the topical corneal penetration of CA. Third, This study investigated the therapeutic effect of CA in the early stage of murine *AF* keratitis. In the future, its long-term safety and efficacy in large animal models need to be evaluated to facilitate its clinical application.

## 5 Conclusions

In summary, our study demonstrates that CA possesses dual antifungal and anti-inflammatory activities in a murine model of *AF* keratitis. It effectively inhibits fungal growth, suppresses excessive inflammatory cell recruitment, and modulates the MyD88/NF-κB signaling pathway. Furthermore, its combination with natamycin shows superior therapeutic efficacy. These findings position CA as a promising lead compound for FK treatment. Future research should focus on elucidating its precise molecular targets in *AF*, optimizing drug delivery systems for topical ocular use, and evaluating its long-term safety and efficacy in larger animal models to facilitate its clinical translation.

## Data Availability

The original contributions presented in the study are included in the article/supplementary material. Further inquiries can be directed to the corresponding author/s.
